# Effect of sarcopenia, osteoporosis, and osteosarcopenia on spine fracture in American adults with prediabetes

**DOI:** 10.3389/fendo.2023.1163029

**Published:** 2023-04-19

**Authors:** Yufang Liu, Sanbao Chai, Xiaomei Zhang

**Affiliations:** Department of Endocrinology, Peking University International Hospital, Beijing, China

**Keywords:** prediabetes, sarcopenia, osteoporosis, spine fracture, osteosarcopenia

## Abstract

**Objective:**

This study aimed to investigate the effect of sarcopenia, osteoporosis, and osteosarcopenia on spine fracture in patients with prediabetes.

**Methods:**

We collected and analyzed the data from the U.S. National Health and Nutrition Examination Surveys during the period from 2009 to 2018. Bone mineral density and the skeletal muscle mass index (SMI) were measured with dual-energy X-ray absorptiometry (DXA). The diagnosis of spine fracture was based on DXA and history.

**Results:**

People with prediabetes were more likely to develop sarcopenia than normal glucose tolerance subjects (OR 1.33, 95% CI 1.07–1.66), while there was no significant increase of osteoporosis in prediabetes (OR 0.91, 95% CI 0.78–1.05). The SMI was independently associated with osteoporosis in prediabetes adults (OR 0.65, 95% CI 0.50–0.85). Both sarcopenia and osteoporosis were positively associated with spine fracture in prediabetes (OR 4.44, 95% CI 1.76–11.21, and OR 2.90, 95% CI 1.85–4.56, respectively). The risk of spine fracture was substantially higher in the presence of osteosarcopenia (OR 6.63; 95% CI, 1.34–32.94) than in the presence of sarcopenia or osteoporosis alone in prediabetes.

**Conclusion:**

In adults with prediabetes, both sarcopenia and osteoporosis are risk factors for spine fracture, and the combination of sarcopenia and osteoporosis further increases the prevalence of spine fracture.

## Introduction

Prediabetes refers to an intermediate metabolic state between normoglycemia and diabetes, and it includes impaired fasting plasma glucose, impaired glucose tolerance, and mildly raised hemoglobin A1c (HbA1c). Although there is no clinically confirmed hyperglycemia in prediabetes, a series of pathophysiological changes related to diabetes have occurred. Recent evidence has shown that the prevalence of diabetes-associated complications in prediabetes starts to rise compared with those with normal glucose levels (1).

Osteoporosis describes a systemic bone disease that is prone to fractures due to a decrease in bone mass and the destruction of bone microstructures, resulting in increased bone fragility, whereas sarcopenia refers to decreased muscle mass, strength, and function. The incidence of both osteoporosis and sarcopenia increases as the aggravated population ages. Both osteoporosis and sarcopenia can lead to a greater risk of falls, fractures, hospitalization, and mortality. Because of the close relationship between the two conditions, the concept of osteosarcopenia has been established, which refers to the coexistence of osteoporosis and sarcopenia.

Previous studies have shown that people with diabetes are more likely to have sarcopenia (2, 3), and people with diabetes are at a higher risk of developing fractures (4). Therefore, sarcopenia and osteoporosis are increasingly recognized as chronic complications of diabetes. For people with prediabetes, the risk of sarcopenia, osteoporosis, and osteosarcopenia and their impact on fractures are still unclear.

In this study, we analyzed the data from the American NHANES from 2009 to 2018 to examine the association among sarcopenia, osteoporosis, and prediabetes and the effect of sarcopenia, osteoporosis, and osteosarcopenia on spine fracture in American adults with prediabetes.

## Methods

### Population

NHANES consists of a cross-sectional multistage, stratified, and clustered probability sample of the deinstitutionalized population in the United States. It was conducted by the National Center for Health Statistics and approved by the National Center for Health Statistics institutional review board. Written informed consent was received for all participants.

The data of participants in the American NHANES 2009–2018 survey were analyzed. NHANES 2009–2018 data are publicly available and can be accessed online (https://www.cdc.gov).

Participants with missing relevant data and the lack of relevant examinations were excluded from the analyses. The analyses of the present study were limited to individuals aged ≥18 years.

### Measurements

Information was collected through family interviews and physical examinations in a mobile examination center. A standardized questionnaire was used to collect data on age, sex, race, education level, physical activity, and the history of fracture. Race was self-reported, and in the present study, it was categorized into white, black, Mexican, Asian, and other races. Current smoking was defined as having smoked 100 cigarettes or more in one’s lifetime and currently smoking cigarettes. The education level was categorized as less than 9th grade, 9–11th grade, high school graduate, AA degree, and college or above. The BMI was calculated by dividing body weight (kg) by the square of height (m). Steroid use was defined as ever taken any prednisone or cortisone pills nearly every day for a month or longer. Information about physical activity was self-reported by participants using the Global Physical Activity Questionnaire since the 2007–2008 cycle. Based on the data from self-reported questionnaire, metabolic equivalents (METs) can be calculated, which were used to estimate the average weekly energy expenditure of participants (5).

BMD was evaluated by DXA. HbA1c levels were measured by testing whole blood samples using the method of high-performance liquid chromatography. The glucose tolerance test is to measure the plasma glucose value 2 h after the oral administration of 75 g glucose.

### Definition of variables

Participants eligible for any of the following conditions were classified as diabetic patients in the present study: (1) a confirmed history of diabetes in questionnaire; (2) HbA1c level ≥ 6.5% (6); and (3) fasting glucose level ≥ 7.0 mmol/L (6). Participants who accord with all of the following conditions were defined as NGT: (1) a denied history of diabetes or prediabetes in the questionnaire; (2) HbA1c level <5.7% (6); and (3) fasting glucose level <5.6mmol/L. The remaining participants were defined as prediabetes.

T-scores were calculated as (BMD_respondent_-mean BMD_reference group_)/SD_reference group_. In the formula above, SD stands for standard deviation. Osteoporosis was defined as a T-score<−2.5 in total lumbar spine (L1–L4) or femoral neck tested by DXA. As recommended by the World Health Organization (7), the diagnosis of osteoporosis should be based on ethnic and sex-specific reference values. Therefore, the race-specific reference value of BMD for the calculation of T-scores at the femoral neck and the lumbar spine was obtained from the Vital and Health Statistics from the Centers for Disease Control (CDC) (8).

The appendicular SMI was calculated by dividing the appendicular skeletal muscle mass (kg) by square of height (m). The SMI cutoff values for the diagnosis of low muscle mass were 7.0 kg/m^2^ for men and 5.5kg/m^2^ for women (9), according to the 2nd meeting of European Working Group on Sarcopenia in Older People.

The presence of either of the following conditions is defined as a spine fracture: previous spine fracture history in the questionnaire; the vertebral fracture status summary in DXA suggests a fracture (mild, moderate, or severe fracture at any level in T4–L4).

### Statistical analysis

The Kolmogorov–Smirnov method was used to evaluate the data distribution. Continuous variables are represented as mean ± standard deviation for normally distributed data or medians and interquartile ranges in parentheses for abnormally distributed data. The chi-square test, Mann–Whitney U test, or independent t-test was performed to compare the differences between two groups when appropriate. Categorical variables are represented as frequency (percentage), and between-group differences were evaluated by the chi-square test. Logistic regression was used to adjust for potential confounding variables when appropriate. *P*-values < 0.05 were considered indicative of statistical significance. All statistical analyses were performed using STATA 12.0.

## Results

The baseline clinical characteristics of the participants enrolled in this study are shown in **Tables 1** (all the subjects) and **2** (subjects with prediabetes). From 2009 to 2018, a total of 23,825 adults were included in the study, of whom 7,427 (31.2%) had prediabetes. As compared to normoglycemic people, subjects with prediabetes had a higher proportion of men, older age, a higher proportion of black race, a lower education level, less physical activity, a higher BMI, and higher waist circumference (WC), so did the subjects with diabetes. In terms of the HbA1c level, as expected, the diabetic group was higher than the prediabetic group, and the prediabetic group was higher than the NGT group. The trend of the insulin level among the three groups appeared the same with the HbA1c level. There was no significant difference among the three groups on Serum 25(OH)D. Interestingly, the lumbar and spinal bone mineral density of the prediabetic group was lower than that of the NGT group, while there was no significant difference between the diabetic group and the NGT group, which may be explained by the excessive weight of the diabetic group (10). In terms of prevalence of osteoporosis, the diabetic group was higher than the prediabetic group, and the prediabetic group was higher than the NGT group. The trend of spine fracture prevalence among three groups appeared the same as osteoporosis prevalence. In terms of the SMI, the diabetic group was higher than the prediabetic group, and the prediabetic group was higher than the NGT group, which may be explained by the excessive weight of prediabetic and diabetic groups.

**Table 1:** Characteristics of U.S. adults with diabetes, with prediabetes and with NGT, 2009–2018.

	NGT (n = 11,896)	Prediabetes (n = 7,427)	Diabetes (n = 4,502)
Sex (male, %)	5,603 (47.1%)	3,654 (49.2%)**	2,314 (51.4%)***
Age (years)	41 ± 18	53 ± 17***	61 ± 14***
Race^a^			
White	4,607 (38.7%)	2,601 (35.0%)***	1,417 (31.5%)***
Black	2,445 (20.6%)	1,829 (24.7%)	1,143 (25.4%)
Hispanic	2,751 (23.1%)	1,806 (24.3%)	1,234 (27.4%)
Asian	1,607 (13.5%)	937 (12.6%)	564 (12.5%)
Other	486 (4.1%)	254 (3.4%)	144 (3.2%)
Educational level^a^			
<9th grade	1,001 (7.0%)	1,011 (11.3%)***	931 (17.0%)***
9–11th grade	1,785 (12.4%)	1,248 (13.9%)	859 (15.7%)
High school	3,172 (22.1%)	2,049 (22.9%)	1,238 (22.6%)
AA degree	4,512 (31.4%)	2,614 (29.2%)	1,513 (27.6%)
College or above	3,890 (27.1%)	2,026 (22.6%)	941 (17.2%)
MET (min/week)	5,040 (2,100, 1,1760)	4,200 (1,680, 10,080) ***	3,360 (1,260, 7,280) ***
BMI (kg/m^2^)	27.4 ± 6.3	30.1 ± 7.2***	32.2 ± 7.6***
WC (cm)	93.6 ± 15.4	101.7 ± 15.9***	108.5 ± 16.2***
HbA1c (%)	5.2 ± 0.3	5.7 ± 0.3***	7.2 ± 1.7***
Insulin (µU/ml)	8.32 (5.58, 12.9)	11.49 (7.15, 18.42)***	13.58 (8.44, 22.62)***
Serum 25(OH)D (nmol/L)	63.7 ± 27.1	64.7 ± 28.0	63.7 ± 27.9
Lumbar spine BMD (g/cm^2^)	1.05 ± 0.15	1.03 ± 0.15***	1.04 ± 0.16
Total spine BMD (g/cm^2^)	1.03 ± 0.14	1.02 ± 0.16**	1.03 ± 0.17
Osteoporosis prevalence (%)	372 (6.39%)	416 (9.72%)***	278 (9.41%)***
SMI (kg/m^2^)	7.7 ± 1.6	8.3 ± 1.8***	8.6 ± 1.8***
Sarcopenia prevalence (%)	824 (6.9%)	245 (3.3%)***	70 (1.6%)***
Spine fracture prevalence (%)	143 (1.2%)	134 (1.8%)***	204 (2.3%)***

**p < 0.01, ***p < 0.001 vs control.

a Data are proportions within group.

MET, metabolic equivalents; BMI, body mass index; WC, waist circumference; 25(OH)D, 25-hydroxyvitamin D.

**Table 2:** Association between prediabetes and the odds of sarcopenia and osteoporosis.

	Sarcopenia			Osteoporosis		
	NGT	Prediabetes		NGT	Prediabetes	
		OR (95%CI)	*P*		OR (95%CI)	*P*
Model 1	1.00 (Ref.)	0.57 (0.49–0.67)	<0.001	1.00 (Ref.)	1.79 (1.59–2.00)	<0.001
Model 2	1.00 (Ref.)	1.33 (1.08–1.64)	0.008	1.00 (Ref.)	0.93 (0.80–1.07)	0.306
Model 3	1.00 (Ref.)	1.33 (1.07–1.66)	0.011	1.00 (Ref.)	0.91 (0.78–1.05)	0.187

Data are summarized as OR (95% CI) unless otherwise indicated.

Model 1 was unadjusted.

Model 2 was adjusted for age, sex, race, and BMI.

Model 3 was adjusted for model 2 adjustments plus current smoking status, educational level and physical activity (MET score).

After adjusting for age, sex, race, BMI, current smoking status, educational level, and physical activity (MET score), people with prediabetes were more likely to develop sarcopenia than NGT subjects (OR 1.33, 95% CI 1.07–1.66), while prediabetes was not an independent risk factor for osteoporosis (OR 0.91, 95% CI 0.78–1.05) (**Table 2**).

Furthermore, in order to explore the effects of osteoporosis and sarcopenia on spinal fractures in the population of prediabetes, we divided the prediabetes population into four groups: normal group (without sarcopenia or osteoporosis), sarcopenia group, osteoporosis group, and osteosarcopenia group (with both sarcopenia and osteoporosis). The subject characteristics of the four groups are shown in **Table 3**. Individuals in the osteosarcopenia group were significantly older and had a lower BMI, lower WC, and less physical activity than normal subjects. As expected, subjects in osteosarcopenia group had lower BMD, a lower SMI, and higher spine fracture prevalence than individuals in the normal group. After adjusting for confounders, the SMI was independently associated with osteoporosis in prediabetes adults (OR 0.65, 95% CI 0.50–0.85) (**Table 4**).

**Table 3:** Characteristics of prediabetes adults with sarcopenia, with osteoporosis, and with osteosarcopenia.

	Sarcopenia (−) Osteoporosis (−)	Sarcopenia (+) Osteoporosis (−)	Sarcopenia (-) Osteoporosis (+)	Sarcopenia (+) Osteoporosis (+)	P-value
	n = 6,339	n = 224	n = 614	n = 21	
Sex (male, %)	3,379 (53.3%)	88 (39.3%)	140 (22.8%)	9 (42.9%)	<0.001
Age (years)	41.2 ± 11.6	43.2 ± 12.5	65.5 ± 12.9	51.4 ± 7.5	<0.001
Race^a^					<0.001
White	1,540 (32.3%)	71 (31.7%)	243 (49.6%)	5 (23.8%)	
Black	1,308 (27.4%)	20 (8.9%)	65 (13.3%)	3 (14.3%)	
Hispanic	1,168 (24.5%)	44 (19.6%)	106 (21.6%)	3 (14.3%)	
Asian	570 (11.9%)	83 (37.1%)	67 (13.7%)	9 (42.9%)	
Other	185 (3.9%)	6 (2.7%)	9 (1.8%)	1 (4.8%)	
Educational level^a^					<0.001
<9th grade	625 (10.1%)	9 (4.3%)	85 (14.0%)	0 (0%)	
9–11th grade	880 (14.2%)	22 (10.4%)	89 (14.6%)	5 (23.8%)	
High school	1,437 (23.2%)	47 (22.3%)	144 (23.7%)	7 (33.3%)	
AA degree	1.844 (29.7%)	64 (30.3%)	167 (27.5%)	3 (14.3%)	
College or above	1,415 (22.8%)	69 (32.7%)	123 (20.2%)	6 (28.6%)	
Steroid use (%)	184 (5.1%)	4 (5.4%)	62(11.8%)	1(6.3%)	<0.001
Current smoker (%)	2,841 (44.8%)	90 (40.2%)	267 (43.5%)	10 (47.6%)	0.513
MET (min/week)	5,880 (2,520, 13,440)	3,360 (1,680, 10,640)	2,520 (11,20, 6,720)	3,360 (1,680, 4,620)	<0.001
BMI (kg/m^2^)	30.8 ± 6.7	21.4 ± 2.4	28.7 ± 6.5	20.4 ± 2.3	<0.001
WC (cm)	101.6 ± 15.3	82.3 ± 8.9	98.9 ± 14.5	82.2 ± 7.4	<0.001
Serum 25(OH)D (nmol/L)	62.0 ± 26.3	61.7 ± 24.8	78.5 ± 30.9	65.2 ± 34.8	<0.001
HbA1c (%)	5.65 ± 0.35	5.53 ± 0.39	5.76 ± 0.30	5.61 ± 0.33	<0.001
Insulin (µU/mL)	12.1 (7.6, 19.2)	6.2 (4.3, 9.9)	10.2 (6.4, 14.3)	5.94 (3.83, 11.86)	<0.001
Lumbar spine BMD(g/cm^2^)	1.04 ± 0.14	0.98 ± 0.12	0.77 ± 0.12	0.78 ± 0.10	<0.001
Total spine BMD (g/cm^2^)	1.06 ± 0.13	0.96 ± 0.11	0.84 ± 0.15	0.78 ± 0.11	<0.001
SMI (kg/m^2^)	8.55 ± 1.65	5.69 ± 0.76	7.96 ± 1.48	5.62 ± 0.89	<0.001
Spine fracture prevalence (%)	108 (1.70%)	7 (3.13%)	47 (7.65%)	2 (9.52%)	<0.001

a Data are proportions within group.

MET, metabolic equivalents; BMI, body mass index; WC, waist circumference.

**Table 4:** Association between the SMI and osteoporosis in prediabetes adults.

	OR (95%CI)	*p*
Model 1	0.79(0.71–0.87)	<0.001
Model 2	0.64(0.49–0.82)	0.001
Model 3	0.65(0.50–0.85)	0.001

Data are summarized as OR (95% CI) unless otherwise indicated.

Model 1 was unadjusted.

Model 2 was adjusted for age, sex, race, and BMI.

Model 3 was adjusted for model 2 adjustments plus current smoking status, educational level and physical activity (MET score).

**Figure 1:**
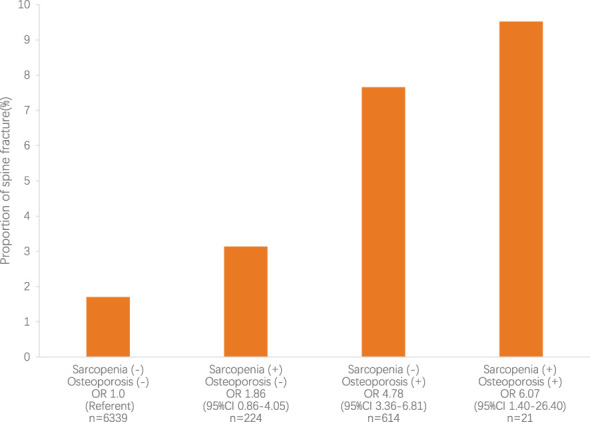
The relative risk for spine fracture according to sarcopenia and osteoporosis status. The relative risk for spine fracture was highest in subjects with sarcopenia and osteoporosis (*P*<0.05). CI, confidence interval; OR, odds ratio.

As shown in **Figure 1**, in the prediabetes population, sarcopenia was not an independent risk factor for spine fracture, while osteoporosis and osteosarcopenia were independent risk factors for spine fracture without adjustment for any confounding factors. In **Table 5**, after adjusting for age, sex, race, BMI, steroid use, current smoking status, educational level, and physical activity (MET score), both sarcopenia and osteoporosis were positively associated with spine fracture in the fully adjusted model (model 3, OR 4.44, 95% CI 1.76–11.21, and OR 2.90, 95% CI 1.85–4.56, respectively). Furthermore, the likelihood of spine fracture was substantially higher in the presence of osteosarcopenia (OR 6.63; 95% CI, 1.34–32.94). Unlike prediabetes, there was no significant association between sarcopenia and spine fracture in the NGT group, while sarcopenia and osteosarcopenia were still positively associated with spine fracture in the NGT group (model 3, OR 2.40, 95% CI 1.53–3.76, and OR 4.31, 95% CI 1.17–15.92, respectively).

**Table 5:** Incidence rate ratios (95% CI) for spine fracture according to categories based on sarcopenia and osteoporosis in NGT/prediabetes adults.

	Sarcopenia (−) Osteoporosis (−)	Sarcopenia (+) Osteoporosis (−)		Sarcopenia (−) Osteoporosis (+)		Sarcopenia (+) Osteoporosis (+)	
		OR (95%CI)	*P*	OR (95%CI)	*P*	OR (95%CI)	*P*
NGT							
Model 1	1.00 (Ref.)	0.47 (0.17–1.27)	0.135	8.87 (6.35–12.39)	<0.001	5.61 (1.72–18.25)	0.004
Model 2	1.00 (Ref.)	1.47 (0.50–4.30)	0.483	2.63 (1.66–4.16)	<0.001	7.87 (2.19–28.24)	0.002
Model 3	1.00 (Ref.)	1.32 (0.44–3.95)	0.620	2.40 (1.53–3.76)	<0.001	4.31 (1.17–15.92)	0.028
Prediabetes							
Model 1	1.00 (Ref.)	1.86 (0.86–4.05)	0.117	4.78 (3.36–6.81)	<0.001	6.07 (1.40–26.40)	0.016
Model 2	1.00 (Ref.)	4.82 (1.99–11.66)	<0.001	2.87 (1.82–4.50)	<0.001	13.00 (2.73–61.93)	0.001
Model 3	1.00 (Ref.)	4.44 (1.76–11.21)	0.002	2.90 (1.85–4.56)	<0.001	6.63 (1.34–32.94)	0.021

Data are summarized as OR (95% CI) unless otherwise indicated.

Model 1 was unadjusted.

Model 2 was adjusted for age, sex, race, and BMI.

Model 3 was adjusted for model 2 adjustments plus steroid use, current smoking status, educational level, and physical activity (MET score).

## Discussion

### Prediabetes and osteoporosis

Recently, the correlation between diabetes and bone health is attracting increasing attention. It is well known that type 2 diabetes predisposes individuals to a higher risk of fractures; even type 2 diabetes is associated with an average or higher BMD (11). Nevertheless, there are few studies exploring the relation between prediabetes and skeletal health, and the results were conflicting (12–14). This study found an increased risk of spine fractures in prediabetes. In addition, in this study, although the risk of spine fracture increased in the prediabetes population compared with the NGT, the prevalence of osteoporosis was not significantly different from that in the NGT population. Previous studies have also found that the risks of hip fractures begun to increase in prediabetes (15). Thus, similar to the condition in diabetes, the bone in prediabetes appears to have relatively low strength for a given BMD. As a result, the BMD as a conventional tool appears to underestimate the risk of fracture in individuals with prediabetes, which is a challenge for clinicians.

### Prediabetes and sarcopenia

A previous study has reported that muscle strength was lower in diabetes patients than individuals without diabetes (16), and type 2 diabetes is related to accelerated loss of leg muscle strength in elderly individuals (17). In fact, type 2 diabetes has already been identified as an independent risk factor for sarcopenia (18). In terms of sarcopenia, it has been revealed that strength of hand grip adjusted by the BMI (19) or body weight (20) is related to prediabetes. Kaga et al. recently reported that prediabetes is an independent risk factor for sarcopenia in older Japanese men but not in older Japanese women (21). In the present study, after adjusting for age, sex, race, BMI, current smoking status, educational level, and physical activity (MET score), prediabetes is an independent risk factor for sarcopenia in the multiracial group. Therefore, it is necessary for healthcare providers to pay more attention to the development of sarcopenia in prediabetes as well as diabetes.

### Sarcopenia and osteoporosis

In the present study, osteoporosis is closely related to the SMI in subjects with prediabetes. To the best of our knowledge, our study is the first attempt to provide the association between the SMI and osteoporosis in U.S. adults with prediabetes. Sharing the same mechanical and molecular mechanisms, muscle and skeleton function are closely linked (22). Both skeleton and muscle mass are intrinsically related to the declined physical performance with aging, while the bone–muscle crosstalk, which is the molecular mechanisms linking bone to muscle function, is less well defined. Hormones were identified as having an important role in the development of osteosarcopenia, including growth hormone (GH)/insulin-like growth factor-1 (IGF-1) and gonadal sex hormones (23).

### Osteosarcopenia and fracture

Previous studies have shown that the coexistence of sarcopenia and osteoporosis was associated with some adverse outcomes, such as depression, malnutrition, peptic ulcer disease, inflammatory arthritis, and reduced mobility (24). Meanwhile, there are studies demonstrating that subjects with both osteoporosis and sarcopenia are at a higher risk of falls and frailty than those with osteoporosis or sarcopenia alone (24, 25). In a Korean study conducted in hip fracture patients, osteosarcopenia was associated with a higher 1-year mortality rate (15.1%) compared with subjects with osteoporosis (5.1%) or sarcopenia (10.3%) alone (26). In the present study, in patients with prediabetes, sarcopenia increases the risk of spinal fractures by 4.4 times, osteoporosis increases the risk of spinal fractures by 2.9 times, and sarcopenia combined with osteoporosis increases the risk of spinal fractures by 6.6 times. As for people with NGT, although sarcopenia does not significantly increase the risk of spinal fractures, its combination with osteoporosis further increases the prevalence of spinal fractures.

In conclusion, patients with prediabetes had an increased risk of sarcopenia compared with people with NGT. In adults with prediabetes, muscle weight loss is associated with osteoporosis; meanwhile, osteoporosis and sarcopenia both increase the risk of spinal fractures, while the combined presence of sarcopenia and osteoporosis further increases the prevalence of spinal fractures. For patients with prediabetes, in order to prevent spinal fracture, attention should be paid to the prevention and treatment of sarcopenia and osteoporosis, and special attention should be paid to the combination of sarcopenia and osteoporosis.

A key strength of this analysis is the source of the data. NHANES is a series of meticulously conducted surveys with continuous quality control, ensuring that the data are timely and of high quality. NHANES also uses population-based cluster random selection to identify a nationally representative sample that can be applied to the whole U.S. population. However, it has some limitations. First, the definition of osteoporosis, in addition to BMD < −2.5, also includes a history of fragility fractures, which were not included in the osteoporosis group because fragility fractures could not be defined. Second, the diagnosis of sarcopenia, in addition to decreased muscle quantity, also includes a decrease in muscle quality, which was not analyzed in this study due to the lack of relevant test results. Third, the reference standards for muscle mass are diverse, and this study uses the criteria of the second meeting of the European Sarcopenia Working Group, which does not necessarily apply to people of African, Asian, Hispanic, or other races. Fourth, because some of the respondents did not complete the full set of examinations, fewer people were diagnosed with osteosarcopenia. It is hoped that data with larger sample size will be available for future studies in this area.

## Data availability statement

The raw data supporting the conclusions of this article will be made available by the authors, without undue reservation.

## Ethics statement

The studies involving human participants were reviewed and approved by National Center for Health Statistics (NCHS) Research Ethics Review Board (ERB). The patients/participants provided their written informed consent to participate in this study.

## Author contributions

YL and SC conceived and designed the experiments. YL performed the data analysis. YL wrote the manuscript. XZ provided supervision. All authors contributed to the article and approved the submitted version.
